# Tincture of Iodine in the Treatment of External Poisoning

**Published:** 1851-07

**Authors:** 


### SURGERY.

*Tincture of Iodine in the Treatment of External Poisoning.*—Dr. T. Smith, of Cincinnati, has used the tincture of iodine, with much success, in several cases of poisoning, the result of contact with the Rhus Toxicodendron (Poison Oak,) Rhus Radicans (Poison Vine,) Rhus Vernx (Swamp Sumac,) &c. Having been frequently called upon to prescribe for this affection, Dr. Smith was lead to the trial of local remedies, likely to prove more prompt than the ordinary antiphlogistic treatment resorted to on such occasions. Within the last two years, he has used the tincture of iodine as a local application, in some half dozen cases, and with such striking good effects, that he confidently recommends it to the profession.—*Abridged from the Western Lancet, May,* 1851.

